# Uterine sparing surgical methods in pelvic organ prolapse

**DOI:** 10.4274/tjod.43179

**Published:** 2015-09-15

**Authors:** Esra Nur Tola, Evrim Erdemoğlu, Ebru Erdemoğlu

**Affiliations:** 1 Süleyman Demirel Univercity Faculty of Medicine, Department of Gyneacology and Obstetrics, Isparta, Turkey; 2 Süleyman Demirel Univercity Faculty of Medicine, Department of Gyneacologic Oncology, Isparta, Turkey; 3 Şifa Hospital, Clinic of Gyneacology and Obstetrics, Isparta, Turkey

**Keywords:** Pelvic organ prolapse, uterine sparing surgery, laparoscopy

## Abstract

Pelvic organ prolapse (POP) is defined as the protrusion of pelvic organs to the vagen and is an important health problem in patients of older age. Today, most women with POP prefer uterine sparing surgery due to the changes in lifestyle, beliefs, pregnancy desire, and understanding the role of the uterus and cervix in sexual function. Therefore, the need for newer surgical procedures that involve less invasive surgery, reduced intraoperative and postoperative risks, and a faster healing time in POP surgery have gained importance. Vaginal, abdominal, laparoscopic, and robotic methods are defined in uterine preserving surgery but there is not yet a consensus on which of them should be chosen. In choosing the proper technique, the patient’s general status, accompanying disease, correct indication, and the surgeon’s experience are all important. In our practice we prefer laparoscopic mesh sacrohysteropexy in patients who prefer to preserve their uterus because of the lower costs and high success rates compared with abdominal and robotic techniques.

## INTRODUCTION

Pelvic organ prolapse (POP) is defined as the protrusion into the vagina of pelvic organs from their normal anatomic position due to ligamentous or fascial support deficiency. The terms uterine prolapse, cystocele, rectocele, and enterocele are used for the identification of localization. Multiple compartmental defects are generally present together. POP is a common health problem that effects half of women aged over 50 years. The risk to women who undergo pelvic reconstruction due to POP and/or urinary incontinence is 11%^(1)^. POP is responsible for 20% of major gynecologic operations in the United Kingdom^(2)^.

Although uterine prolapse generally presents incidentally, it may cause symptoms that disrupt quality of life, such as a sense of pressure and discomfort. Symptoms include protrusion from the vagina, pain in the lower abdomen and hip, the feeling of vaginal fullness, and regression of the symptoms when lying down. There may be irritation or infection of the skin when the uterus is completely protruded^(2)^. Most cases of uterine prolapse have a higher incidence of miction and urinary incontinence associated with cystocele, difficulty in defecation, and rectal fullness associated with rectocele, as well as sexual dysfunction due to anterior and posterior wall defects.

POP treatment changes according to the degree of the prolapse, symptoms, patient’s general status and choice, activity level and fertility desire, and the surgeon’s choice of procedures and skill^(1)^. Observation is appropriate for asymptomatic or mildly symptomatic patients. Women with severe prolapse or symptoms require treatment, the strategies of which may be divided into two parts: medical or surgical. Medical approaches include the use of pessary and pelvic floor exercises,^(1)^ it has been defined ‘uterine sparing methods’ or ‘not’ in surgical treatment.

Vaginal hysterectomy has been performed to treat prolapse for many years, but it has been found to be inadequate for the elimination of pelvic support deficiency, because prolapse is not the cause, but the result^(3)^. When vaginal hysterectomy performed because of benign etiologies were compared with those performed for the treatment of prolapse, the occurrence of apical prolapse was observed to be six times higher^(4)^. Uterine-sparing surgery is increasingly preferred in the treatment of POP surgery following the field’s increased understanding of the benefits of the uterus.

## UTERINE SPARING SURGICAL MODALITIES

Today, most women with POP prefer uterine-sparing surgery because of changes in lifestyle, the desire for pregnancy, and the understanding of the role of the uterus and the cervix in sexual function. In a study in which women were asked whether they would prefer to preserve their uterus, 20-46% said they would choose preservation^(5)^. In that study, as educational level increased, more women preferred to keep their uterus. It has been reported that women who have undergone hysterectomy feel less feminine^(6)^; hysterectomy can affect both sexual life and personality^(7)^. Uterine contractions, which help to reach orgasm; shortening of the vagina during hysterectomy; nerve damage; and loss of self-esteem may all affect one’s sexual life. In conclusion, the patient’s self-esteem, body image, and sexuality all increase with uterine-sparing procedures^(8)^.

The continued risk of cervical and endometrial cancer may be a disadvantage of uterine-preserving POP surgery^(9)^. Therefore, cervical screening programmes should be continued as usual after uterine-sparing procedures have been performed. Beyond the decreased risk of perioperative and postoperative complications, the risk of cervical and endometrial cancer may be ignored, however^(9)^.

In uterus-sparing surgery, the pelvic anatomy is not altered, there is less intraoperative bleeding, and operating times and hospital length of stay are both reduced^(10)^. As a result, the need for newer surgical procedures for POP surgery has gained importance. These newer procedures involve less invasive surgery, reduced intraoperative and postoperative risks, and faster healing times. Vaginal, abdominal, laparoscopic, and robotic methods have been defined in uterine-sparing surgery,^(7,8,9,10,11)^ but there is not yet a consensus on which method is best. There is difficulty in performing similar surgical techniques because there is no correlation between the size of the anatomic defect and the symptoms of the prolapsus, as well as the inadequacy in identifying patients with genital prolapse^(2)^. In choosing the proper technique to use, the patient’s general status, any accompanying disease, accurate indication, and surgeon’s experience are all important factors.

## TRANSVAGINAL APPROACHES

ive techniques have been described for uterine-sparing transvaginal surgery: the Manchester operation, uterosacral ligament fixation, sacrospinous ligament fixation, iliococcygeal suspension, and colpocleisis. Nicita et al.^(12)^ reported no recurrence in a 31-month follow-up of patients who had undergone vaginal-approach uterine-sparing surgery. In another transvaginal approach study, the success rate was reported to be 89.5% over a 19-month follow-up period^(13)^.

The Manchester procedure, the oldest method, was first described in 1888 by Archibald Donald of Manchester, England, for the treatment of cervical elongation^(7)^. The procedure involves amputation of the cervix, cardinal ligament plication, uterosacral ligament plication, and anterior and posterior colporrhaphy^(2)^. This operation has the disadvantages of obstetric complications such as subfertility, infertility, prolonged labour, pregnancy loss, high failure and recurrence rates, dyspareunia, dysmenorrhoea, and difficulty in endometrial and cervical sampling due to cervical stenosis^(7)^. This operation type is often not preferred because of these issues^(8)^.

In transvaginal uterosacral plication, the peritoneal cavity is entered through a posterior colpotomy and the uterosacral ligaments are divided from the cervix, plicated in the midline, and reinserted into the cervix. In this way, the cardinal ligaments pull the cervix upwards into the midline^(14)^. Ureteral injury and neurologic morbidity are high in this type of surgery because the ureteral dissection cannot be made adequately^(7)^. Another disadvantage of this method is that the plication proportion is low because the uterosacral ligaments are transected close to the uterus.

In sacrospinous ligament fixation, the cervix and uterosacral ligament are fixed 2 cm medial to the ischial spine, preferentially to the right sacrospinous ligament. When compared with vaginal hysterectomy, it is a safe and effective method^(15)^. There may be postoperative hip pain and bleeding in 10-15% of patients with sacrospinous hysteropexy because of nerve damage around the sacral plexus and the pudendal nerve^(15)^. Hefni et al.^(16)^ found no difference between recurrence rates when comparing groups in which the uterus was spared or not spared. Van Brummen et al.^(17)^ found similar recurrence rates in their study, in which they compared sacrospinous hysteropexy and vaginal hysteropexy. In uterine-sparing sacrospinous fixation cases, recurrence rates have been reported to be very low^(18)^. The disadvantage of this technique is the deterioration of the vaginal axis, because the sacrospinous ligament fixation is performed unilaterally.

## TRANSVAGINAL MESH SURGERY

Uterine sparing POP surgery can be performed transvaginally using meshes. However, because biologic grafts can fail, the use of synthetic mesh has become more popular. Meshes can be applied separately or with the help of a mesh kit. Total surgical kits (new-generation meshes Prolift, Gynecare, Johnson & Johnson, New Jersey, USA; Avaulta Anterior BioSynthetic Support System) have the advantage of easy application and short operating times, but the disadvantage of a high rate of erosion^(19)^. In a recent study in which these new-generation meshes were used, 98% of patients had no intraoperative complications, 0.62% had bladder injury, mesh erosion in 4%, and patients had remarkable regression in their vaginal symptoms during follow-up^(20)^. Mesh-augmented POP surgery has better anatomic outcomes, but similar functional outcomes; mesh surgery is inappropriate and selected patients may reduce the mesh complication rates.

## LAPAROSCOPIC APPROACHES

The laparoscopic approach to POP surgery is technically difficult. The surgeon has to be very familiar witgh laparoscopic suture techniques, pelvic anatomy, and retroperitoneal space anatomy. Laparoscopic techniques include laparoscopic suture sacrohysteropexy (LSH) and laparoscopic mesh sacrohysteropexy (LMH)^(7).^

## SUTURE SACROHYSTEROPEXY

LSH is a safe and effective procedure in the management of POP for those who want to spare their uterus. In this procedure, the pouch of Douglas is closed, and the uterosacral ligaments are plicated and attached to the cervix. In a study in which 43 patients with symptomatic uterine prolapsus who had been treated with LSH were followed up for 12+47 months and prolapsus symptoms regressed in 81% of patients and disappeared in 79%; LSH was reported to be a safe and effective method in prolapsus surgery^(21)^. The success rate of LSH seems to be lower than that of LSH, which is performed with meshes. There is less ureter injury and uterosacral ligament plication is more successful because ureter dissection can be performed better in LSH, which leads to better results in POP surgery. In addition, because abdominal exploration is performed laparoscopically, co-existing pathologies can be restored during the same session.

## MESH SACROHYSTEROPEXY

In LMH, the uterus is suspended from the sacral promontorium using a non-absorbable synthetic mesh at the level of the uterosacral ligament^(2)^. By this suspension, the elevation of the vaginal axis restores the problem of anterior vaginal wall prolapse (cystocele), long-term anatomic restoration is achieved, and a normal vaginal axis and sexual function are maintained. This shows that sacrohysteropexy restores vaginal axis, is safe, and improves sexual function and micturition^(10)^. Ideally, the mesh used in sacrohysteropexy must be inert, resistant to mechanical stress, sterile, and non-carcinogenic.

Laparoscopic sacrohysteropexy (LS) has become a preferred method because surgeons’ experience and skills, augmented by improved laparoscopic equipment, have developed^(7)^. Healing times are shorter, the anatomy is more visible with laparoscopy, hemostasis is much better, and blood loss is less because of sufficient pressure. The other advantages are that postoperative pain is less, hospitalization times are shorter, the healing process is faster, incisions are smaller, ureteric injury is less than with the vaginal route, and it has positive effects on sexual function due to maintenance of the vaginal anatomy^(2)^. In addition, there are fewer intraoperative adhesions, which prevents future infertility. Price et al.,^(7)^ in a 10-week postoperative follow-up of 511 patients, found the success rate to be 98%, and reported that this was an effective and feasible procedure for treatment of POP. Rosenblatt et al.^(22)^ showed that this was an effective and safe method in their study of 40 patients. Perez et al.^(23)^ reported that life quality increased after laparoscopic sacrocolpopexy.

Complications are rare in laparoscopic sacrohysteropexy. Probable complications include retroperitoneal hematoma, which can occur during the peritoneal dissection over the sacral promontorium (2%); large bowel injury (2%); mesh erosion; and recurrence^(2)^. The most common approach in LS is to place the proximal portion of the mesh on the posterior cervix and upper vagina, and the distal portion on the sacrum. These who have anterior prolapsus may not benefit from this method; in these cases, some authors have recommended passing the arms of the mesh from the posterior to anterior cervix and vagina through the broad ligament. However, uterine vasculature may be constricted and uterine vascular supply may be damaged, which may be important in uterine expansion during pregnancy. Vree et al.^(24)^ fixed the arms of the mesh to the medial of the uterine arteries at the level of internal os.

If there are no hysterectomy indications, LS seems to be a good method for the treatment of POP. Correct placement of the mesh plays an important role in long-term success. Posterior located myoma uteri is not a contraindication for laparoscopic sacrohysteropexy. Faraj et al.^(2)^ successfully performed laparoscopic myomectomy and sacrohysteropexy in a patient aged 55 years with grade 3 uterine prolapse and intramural myoma in the uterosacral ligament that did not allow for mesh placement.

## ABDOMINAL TECHNIQUES

### Abdominal sacrohystreopexy

Abdominal sacrohysteropexy (ASH) has been considered to be the gold standard for uterine prolapse because of its high success rates and long-term results^(25)^. In ASH, with the help of a mesh, the uterus is suspended from the sacral promontorium at the level of the uterosacral ligament. The abdominal approach in uterine sparing POP surgery has been found to achieve a success rate of 100% in apical compartment defects and 80% in other defects^(26)^. Barranger et al.,^(27)^ in an 8-160-month (mean 44.5) follow-up of 30 patients upon whom they had performed Burch and posterior colporrhaphy in the same session, reported intraoperative complication rates as 6.6% and postoperative complication rates as 13.3%. In the same study, mesh erosion was reported to be 3.3%, recurrence 6.6%, and 93.3% of post-operation pregnancy cases ended successfully. In their 25-month follow-up studies of patients after abdominal sacrohysteropexy, Leron et al.^(28)^ (in their 20 cases) and Demirci et al.^(29)^ (in their 13 cases) reported no intraoperative or postoperative complications; both studies reported the recurrence risk as <5%^(28,29)^. In patients with POP who want to spare their uterus, ASH provides long-term anatomic restoration and normal vaginal axis, is safe and effective, and has high success rates and low complication rates.

However, ASH does have gastro-intestinal complications such as bowel injury; ileus; retroperitoneal hematoma; wound-site infection (11.1%); small bowel obstruction (18.9%); and general complications such as mesh erosion, hemorrhage (8.9%), and the recurrence of prolapses^(11,15,27,29)^. Moiety et al.,^(11)^ in their study of 33 patients who underwent ASH, reported the mean operation time as 45.7 minutes (range, 30-75 minutes) and hospitalization time as 2.45 days. They reported one rectal injury, one median sacral venous injury, two voiding difficulties, and two recurrences in sixth months. They reported the objective success rate as 93.3% and the subjective success rate as 83.3%, and also that the technique was safe, successful, and easy to learn^(11)^.

Constantini et al.(26) performed ASH on 47 patients and LS on 8 patients among 55 patients with symptomatic POP and followed them up for an average of 63 months^(26)^. There were no cases of prolapse recurrence, but they found cystocele in 7.7%, rectocele in 5.7%, and anterior and posterior vaginal wall prolapsus in 13.4% of the patients. They observed significant improvement in their patients’ sexual-, urinary-, and bowel symptoms^(26)^. Operation times are short, morbidity is more infrequent, the technique is easier, and success rates are high with the abdominal approach, but the risk of mesh erosion is higher than in laparoscopic treatment.

## ROBOTIC SURGERY

### Robotic assisted laparoscopic sacrohysteropexy

Robotic-assisted laparoscopic sacrohysteropexy (RALS) allows 3D viewing, suturing and dissection capacity, has increased robotic manoeuvring capability, and is minimally invasive. It provides good hemostasis, shorter hospitalization times, less morbidity and postoperative pain, but it is an expensive method^(30)^. Seror et al.^(31)^ reported that better long-term results could be achieved with improved suturing and dissection. The long-term effects of RALS have been found to be comparable with abdominal operations^(25)^. In two studies, intraoperative bleeding was found to be less than 50 mL^(32,33)^. Geller et al.^(30)^ reported intraoperative bleeding to be 103 mL in RALS and 412 mL with abdominal sacropexy. The hospitalization time was reported to be one day by Geller et al., and two days by Mouruks et al.^(32)^; Geller et al.^(30)^ reported legth of stay was three days for abdominal sacrohysteropexy and Mouriks et al. reported that RALS increased quality of life, and was a safe and effective method^(32)^.

The mean operation time in RALS is 160-328 minutes^(30,32,33,34)^. The operating time in ASH is 89 minutes, and LS ranges from 180-237 minutes^(33,35)^. RALS and LS require much time and practise to be learned^(34)^ Operation times in LS decrease after the first 30 procedures, and stabilize after 90 procedures^(35)^.

### Pregnancy after uterine sparing surgery

Pregnancy data after uterine sparing POP surgery are not clear. Women who have prolapsus surgery must be warned because the effects of pregnancy and delivery with this reconstructive procedure are not clear. Twenty-four (9.4%) of 257 women who underwent POP surgery became pregnant, of these, six had Cesarean births, and ten had vaginal births, eight of which resulted in abortion^(1)^ Lewis et al.^(36)^ reported mesh-related pelvic pain in the third trimester and found recurrence two years after delivery in a woman who became pregnant six months after undergoing laparoscopic sacrohysteropexy. She was then treated with robotic supracervical hysterectomy, sacrocolpopexy, and perineorrhaphy^(36)^.

### The outcomes of uterine sparing surgery

Several studies have been conducted to determine whether recurrence rates increase after uterine sparing surgery compared with procedures that do not spare the uterus. Hefni et al.^(16)^ found no difference between recurrence rates. Brummen et al.^(17)^ compared recurrence rates between sacrospinous hysteropexy and vaginal hysteropexy and found similar recurrence rates. Recurrence after sacrospinous fixation, which spares the uterus, have also been reported to be low^(18,37)^. Nicita et al.^(12)^ reported no recurrence in a 31-month follow-up study after vaginal-approach uterus-sparing surgery.

## RESULTS

Uterine-sparing techniques in POP surgery have the advantage of not disturbing the pelvic anatomy, less intraoperative bleeding, and shorter operation and hospitalization times. These techniques also maintain the patient’s self-esteem and sexuality. Uterine-sparing POP surgery may be performed using abdominal, laparoscopic, and robotic methods. LMH is a successful and safe method of POP surgery. In our practise, we prefer LMH in patients who prefer to spare their uterus because of the lower costs and higher success rates compared with abdominal and robotic techniques.
